# Activity-related pain predicts pain and functional outcomes in people with knee osteoarthritis: A longitudinal study

**DOI:** 10.3389/fpain.2022.1082252

**Published:** 2023-01-13

**Authors:** Mark Overton, Nicola Swain, Carrie Falling, David Gwynne-Jones, Roger Fillingim, Ramakrishnan Mani

**Affiliations:** ^1^ Centre for Health, Activity and Rehabilitation Research, School of Physiotherapy, University of Otago, Dunedin, New Zealand; ^2^ Department of Surgical Sciences, Otago School of Medicine, University of Otago, Dunedin, New Zealand; ^3^ Pain Research and Intervention Center of Excellence (PRICE), Department of Community Dentistry and Behavioural Science, University of Florida, Gainesville, FL, United States

**Keywords:** knee osteoarthritis, pain, sensitization, activity-related pain, quantitative sensory testing, longitudinal

## Abstract

Knee Osteoarthritis (OA) is a prevalent musculoskeletal condition, commonly resulting in pain and disability. However, pain and disability in this population are poorly related with the degree of structural joint damage. Underlying pain mechanisms, including activity-related pain and sensitization assessed *via* Quantitative Sensory Testing (QST), may better predict pain and functional outcomes of those with knee OA. Therefore, the aim of this study was to explore whether activity-related pain and sensitization assessed *via* QST predict future pain, function, fatigue, physical performance and quality of life outcomes in those living in the community with knee OA. Eighty-six participants with knee OA were recruited in Dunedin, New Zealand. Those eligible to participate underwent baseline testing including QST as well as measures of activity-related pain including Movement-evoked Pain (MEP) and Sensitivity to Physical Activity (SPA). Outcome measures exploring pain, function, fatigue and quality of life outcomes were collected at baseline, and two follow-up periods (two and nine weeks). Univariable linear regression models were developed followed by multivariable linear regression models for each prognostic marker adjusting for age, gender, BMI, OA duration, baseline pain intensity and socioeconomic status. Activity-related measures of pain, including MEP and SPA, demonstrated predictive associations with pain and functional outcomes prospectively in those with knee OA. Therefore, those demonstrating activity-related pain are at future risk of greater pain, disability and reduced quality of life. Larger, externally validated longitudinal studies are required which include individuals with more severe knee OA.

## 1. Introduction

Knee osteoarthritis (OA) is among the most prevalent conditions globally, contributing to significant societal costs and years lived with disability (1–3). Chronic pain is a common symptom of knee OA which is often attributed to changes in joint structures (4). However, recent literature highlights an apparent discordance between pain, disability and the degree of structural changes observed in the knee joint (4, 5). Underlying pain mechanisms including nervous system sensitization have emerged as important considerations in musculoskeletal and pain research over recent decades. Sensitization, which is defined as “increased responsiveness of nociceptive neurons to their normal input, and/or recruitment of a response to normally subthreshold inputs,” (6) has been shown to play a significant role in contributing to pain and disability in the knee OA population (7–9).

Assessment of sensitization in knee OA through Quantitative Sensory Testing (QST) has demonstrated a distinct subgroup of individuals with peripheral and central nervous system sensitization (10, 11). In a study investigating sensitization in those with knee OA, 71% were found to have at least one abnormality on QST, with many displaying widespread hyperalgesia and heightened mechanical temporal summation in comparison to healthy individuals (10, 12). Recent prospective studies have explored whether these QST abnormalities predict future outcomes in people with knee OA. These studies found that those with higher levels of sensitization demonstrated on QST were less responsive to analgesic medications and at risk of poorer surgical outcomes across a range of timeframes (13–21). Fewer observational studies have used QST measures to predict knee OA symptoms. One prospective study reported that greater temporal summation of pain predicted the severity of knee OA pain over the ensuing month among non-Hispanic White but not non-Hispanic Black individuals. Notably, this study only included a single QST measure and had a short follow-up period (22). Studies that explore a greater range of sensitization measures as predictors of pain and functional outcomes in a community sample of those self-managing their knee OA are yet to be performed. Better identifying those at risk of poorer outcomes in the community could assist in deciding who would best benefit from treatments specifically targeting the underlying pain mechanisms.

People with knee OA often experience pain during functional activities such as walking, crouching and climbing stairs (23). Activity-related pain measures, such as Movement-evoked Pain (MEP) and Sensitivity to Physical Activity (SPA) have been used to capture information on distinct aspects of activity-related pain (23–25). Furthermore, as pain information is captured during functional tasks, these measures likely provide greater ecological validity to living and functioning with knee OA in comparison to lab- and clinic-based tests (24, 26–28). Recent studies have confirmed that activity-related pain demonstrates predictive associations with important outcomes. These showed that SPA cross-sectionally predicts levels of function in those with knee OA (29, 30). Additionally, a recent study showed that greater MEP prior to a total knee arthroplasty (TKA), predicted greater post-operative pain at one year (31).

Studies exploring mechanisms of knee OA pain have assessed the relationship between SPA and MEP, and measures of underlying pain mechanisms assessed *via* QST. Both MEP and SPA have demonstrated statistically significant relationships with temporal summation, a marker of ascending facilitation and sensitization processes within the central nervous system (30, 32). Therefore, activity-related pain measures such as MEP and SPA may be more clinically feasible measures of sensitization that do not rely on specialist equipment or training, unlike QST. However, prior to activity-related pain measures being routinely adopted in clinical practice, studies are required to explore the predictive capacity of SPA and MEP in a community sample of those with knee OA. By identifying those at greater risk of worse outcomes, tailored and timely multidisciplinary interventions could be implemented for those in greatest need.

To the best of our knowledge, this is the first study to explore whether assessments of nervous system sensitivity, including comprehensive QST and activity-related pain measures, prospectively predict pain and functional outcomes in people with knee OA who live in the community. Exploring whether sensitization predicts pain outcomes may contribute to developing a better understanding of the different factors contributing to the pain experience in those with knee OA. Therefore, the current study aims to explore whether QST and activity-related measures of pain, predict future pain, fatigue, disability, physical performance, and quality of life outcomes in people with knee OA in New Zealand. It is anticipated that those demonstrating greater pain sensitization on QST and activity-related pain measures, will demonstrate worse pain and functional outcomes prospectively.

## 2. Materials and methods

### 2.1. Study design

**Figure 1:**
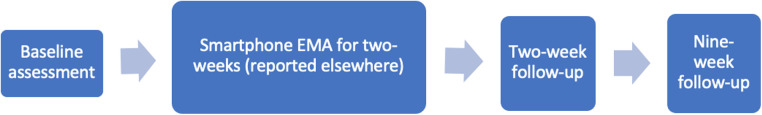
Overview of stages in the U-KOPE study.

Understanding Knee Osteoarthritis Pain Experiences (U-KOPE) is a prospective longitudinal study including smartphone Ecological Momentary Assessment (EMA) completed in Dunedin, New Zealand. This investigation reports predictive associations between baseline measures of sensitization and validated patient-reported outcome measures collected at two-week and nine-week follow-ups. A summary of the current study is presented in Figure 1. This study was developed in consultation with the CHecklist for critical Appraisal and data extraction for systematic Reviews of prediction Modelling Studies (CHARMS) (33, 34). Ethical approval was obtained through the New Zealand Central Health and Disability Ethics Committee (21/CEN/89). Cultural consultation was sought through the Ngāi Tahu Research Consultation Committee.

### 2.2. Participants

Participants were eligible for inclusion if aged 45–85 years, reported a diagnosis of knee OA and experienced knee pain on most days for at least three months. Participants fulfilling NICE guidelines for a clinical diagnosis of knee OA were also included (>45 years of age, activity-related pain and morning stiffness lasting no longer than 30 min) (35).

Participants were excluded if they were non-English speaking, had an autoimmune condition or other forms of inflammatory arthritis, had uncontrolled hypertension, skin conditions, lower limb sensory loss, were pregnant or within six months postpartum, had undergone or were scheduled for total knee arthroplasty, were recovering from a separate lower limb injury, had a neurological condition, impaired cognition or psychiatric illness (excluding stress, anxiety or depression).

Participants were recruited from Dunedin, New Zealand within hospital outpatient settings and the community through advertisements in local newspapers, health practices and online. Participants were given a $100 voucher to recognise any costs involved with participating.

### 2.3. Assessment

Eligible participants attended a 90-minute baseline assessment and two 30-minute follow-up assessments (weeks two and nine following baseline assessment) at the University of Otago. All participants completed reliable and validated questionnaires (Table 1) for measuring biopsychosocial constructs involved in pain and disability in the knee OA population (69, 74, 94). Selected outcome measures are recommended by Outcome Measures in Rheumatology (OMERACT), Osteoarthritis Research Society International (OARSI) and the Initiative on Methods, Measurement, and Pain Assessment in Clinical Trials (IMMPACT) for assessing those with painful knee OA (96, 97).

**Table 1:** Baseline assessment measures.

Demographics and anthropometrics measures	Pain and function measures	Sensitization measures
Age Gender Ethnicity Education level Work status Residential address Socioeconomic status Body Mass Index (36, 37) Hip to Waist Ratio (36, 37) Quadriceps Strength (38–40) Montreal Cognitive Assessment (41)	Brief Pain Inventory – Short Form (42, 43) Widespread Pain Sites (44, 45) Measure of Intermittent and Constant Osteoarthritis Pain – Knee (46) Knee Injury and Osteoarthritis Outcome Score (47, 48)	Punctate Pain Intensity (49, 50) Cold Pain Intensity (49, 51, 52) Pressure Pain Threshold (9, 11, 53–66) Mechanical Temporal Summation (22, 55, 67) Conditioned Pain Modulation (66–68) Sensitivity to Physical Activity (29, 30, 69–71) Movement-evoked Pain (28)
Psychosocial Measures	Health and Lifestyle Measures	
Depression, Anxiety, Stress Scale-21 (72, 73) Pain Catastrophising Scale (74–78) Pain Self-Efficacy Questionnaire-2 (79, 80) Knee Osteoarthritis Fears and Beliefs Questionnaire (81). Brief Coping Strategies Questionnaire (82) Social Support and Pain Questionnaire (83)	Current Medications (84) Modified Charleston Comorbidity Index (mCCI) (85, 86) Pittsburgh Sleep Quality Index (PSQI) (74, 87–90) International Physical Activity Questionnaire – 7-day Short Form (91–93) 12-item Short Form Survey (94, 95)	

#### 2.3.1. Demographic measures

Participant characteristics, including demographic information (age, sex, ethnicity, educational level, residential address, and work status), and anthropometrics (height, weight, hip and waist circumference) were recorded to calculate Body Mass Index (BMI) (kg/m^2^) and hip-waist ratio (36, 37). The Montreal Cognitive Assessment (MoCA), a highly reliable and valid tool for detecting mild cognitive impairment, was administered to participants at the beginning of their baseline assessment (41). In those scoring less than 16 on the MoCA, testing was discontinued and these participants were excluded from the study (98). Quadricep strength testing was performed as described in previous studies using a handheld dynamometer (Lafayette Hand-held Dynamometer, Lafayette Instrument Evaluation, Lafayette, Indiana, USA) (38, 39). This method demonstrates excellent test-retest reliability and validity in people with knee OA (38–40).

#### 2.3.2. Pain and function measures

Pain intensity and interference were determined for the affected knee using the short-form Brief Pain Inventory (BPI) (99). The short-form BPI has been validated for use in the arthritis population (42, 43). The BPI body chart asked participants to indicate body regions where they experienced pain. Selected regions were summed to calculate the number of widespread pain sites (44, 45). Widespread pain has been linked with alterations in central pain mechanisms (100, 101).

A Measure of Intermittent and Constant Osteoarthritis Pain (ICOAP): KNEE, was used to determine constant and intermittent pain experiences which have been associated with sensitization in knee OA (46, 102). Preliminary psychometric testing suggests the ICOAP is a valid and reliable pain measure for OA when compared with the Knee Injury and Osteoarthritis Outcome Score (KOOS) and Western Ontario and McMaster Universities Osteoarthritis Index (WOMAC) (46). The KOOS was also used to measure pain, symptoms and functioning associated with knee OA (94). The KOOS demonstrates adequate measurement properties in the knee OA population (47, 48).

#### 2.3.3. Measures of sensitization

The following procedures were used to quantify aspects of sensitization.

1. **Quantitative Sensory Testing.** Established and standardised QST assessment procedures were completed and are described in more detail below. These QST procedures demonstrated acceptable psychometric properties when used to assess abnormal somatosensory processing in those with knee pain (103). “Bedside” QST procedures were also completed, with these measures being highly correlated with laboratory-based QST (104). The following QST measures were performed:
  - **Cold pain intensity (CPI)** was assessed using an ice cube which was placed on the participant's non-dominant wrist followed by the affected knee for 10 s each. Immediately following each trial, participants reported their greatest pain intensity on a 101-point NPRS (Numeric Pain Rating Scale) (0 = no pain, 100 = worst pain imaginable) (51). Two trials at each site were performed with the average being calculated. This bedside QST procedure is valid and reliable, significantly correlating with laboratory-based testing (49, 51, 52).
  - **Pressure Pain Threshold (PPT)** was measured at the affected medial knee, tibialis anterior (TA) (5 cm below the tibial tuberosity) and at the non-dominant wrist (9, 11, 53–66). A handheld pressure algometer with a probe area of 1 cm^2^ was used at a ramp of 50 kilopascals (kPa) per second (53, 54, 66). Participants were asked to indicate the moment that deep pressure became painful. The average across three trials was calculated (54, 66, 105). Up to five trials were completed at each site if outlier readings were collected.
  - **Punctate Pain Intensity (PPI):** PPI was assessed using a 300-gram nylon monofilament over the patella of the affected knee and at the non-dominant wrist as performed by previous studies (49–51). The nylon monofilament was applied perpendicular to the skin at each testing site with enough force to bend the filament. Immediately following each trial, participants reported their pain intensity on a 101-point NRPS. The average across three separate trials was calculated.
  - **Mechanical Temporal Summation (MTS)** was assessed over the patella of the affected knee and at the non-dominant wrist (55). Pain ratings using a 101-point NPRS were recorded following a single 300-gram nylon monofilament stimulus. Subsequently, 10 consecutive stimuli were applied at a rate of one stimulus per second within a 1 cm^2^ area of skin. After the final stimuli, participants reported their peak pain intensity. The difference between the first pain rating and the peak pain rating was used to calculate a “wind-up” ratio. Three separate trials were performed at each site. This method has been used to calculate MTS in previous studies with greater MTS representing a marker of sensitization within the central nervous system and associated with greater pain intensity (22, 55, 67). Those reporting a ≥20/100 NPRS change, representing the minimally clinically important difference for pain intensity in those with knee OA would be classified as demonstrating MTS (106). Continuous MTS scores were used in statistical models.
  - **Conditioned Pain Modulation (CPM)**, a measure of descending pain modulation, was examined as per previous studies (11, 57, 61, 66–68, 107):
    ○ Conditioning stimulus: Participants were asked to submerge their dominant hand in a manually circulated 10-degree Celsius ice bath for two minutes or until intolerance (67, 68).
    ○ Test stimulus: PPT40 (PPT with participants indicating when their pain reaches an intensity of 40/100 on the NPRS) was assessed at the non-dominant forearm before and at 30, 60 and 90 s following the conditioning stimulus.

For descriptive purposes, participants were classified as being facilitators, inhibitors or non-responders based on the calculated standard error of measurement (108). A change >2 SEM was interpreted as inhibition and <2 SEM as facilitation. Those with change scores <±2 SEM were categorized as non-responders (108). Continuous CPM scores were used in statistical models.

2. **Activity-related pain:** Performance-based tests included a Six-Minute Walk Test and a 30-second Chair Test which have been used in previous studies exploring activity-related pain (30, 69). Discomfort ratings were collected on a 101-point discomfort rating scale (0 = no discomfort, 100 = extreme discomfort) before, during and after each test (30). Temporal summation related to Sensitivity to Physical Activity (SPA) was calculated as the difference between prior and peak discomfort ratings to provide a “wind-up ratio.” Those reporting a 20-point increase in discomfort ratings were deemed to have SPA (109). SPA provides a more ecologically valid measure of temporal summation and has been linked with central sensitization and predicting greater pain intensity and reduced functioning (29, 30, 70, 71). **Movement-evoked pain (MEP)**, representing the average level of pain experienced while undergoing performance-based testing was calculated by taking the average of the discomfort ratings across the 6-Minute Walk Test (28, 32). MEP, therefore, represents the average pain experienced during testing, while SPA represents the change in pain during testing, each potentially having distinct mechanisms.

#### 2.3.4. Psychosocial, health and lifestyle measures

Valid and reliable measures used for assessing psychosocial as well as health and lifestyle-related factors are presented in Table 1.

### 2.4. Data collection and analysis

#### 2.4.1. Sample size calculation

The primary analysis in the present study included multivariable regression models adjusting for age, sex, socioeconomic status, BMI, baseline pain intensity and disease duration. A sample size of 81 was calculated using G*Power 3.1 based on a 0.25 effect size, power of 0.9 and error of 0.05 with seven predictors (110). A final sample of 101 participants was recruited to account for a 20% dropout rate.

#### 2.4.2. Data collection

Sensitization data analysis and questionnaire scoring were not performed until all participants completed the study.

#### 2.4.3. Statistical methods

All statistical analyses were conducted using SPSS (Version 28.0.1.0). Both dependent and independent variables were assessed for normality by exploring Kolmogorov-Smirnov tests, Shapiro-Wilk tests as well as skew and kurtosis. Non-normally distributed data underwent logarithmic, square root and inverse transformation, with most unable to be transformed to the normal distribution.

Independent variables included PPT's, PPI, MTS, SPA, MEP, CPM, CPI and number of pain sites. Baseline dependent variables included the BPI, KOOS, ICOAP, 6 MWT distance, 30 sCST number and SF-12 outcomes. Longitudinal dependent variables included the BPI, KOOS, Brief Fatigue Inventory (BFI), SF-12 and Keele Assessment of Participation (KAP). Models were developed for each dependent variable including one predictor variable alongside covariates. Univariable and multivariable regression analyses were completed to explore the cross-sectional and prospective predictive associations between measures of sensitization and knee OA outcomes. Non-transformed raw data was used as indicated by the Gauss-Markov theorem (111, 112).

Univariable regression was first completed with *p*-values ≤0.2 allowing predictors entry into the multivariable regression analysis. Bivariate correlations were also collected within univariable regression analyses. The following ranges were used to classify effect sizes to interpret the strength of the relationship: zero to 0.25 – small; 0.25 to 0.5 – fair; 0.5 to 0.75 – moderate to good; and >0.75 – good to excellent (113).

Multiple adjusted linear regressions each including one sensitization predictor were then completed for each of the dependent variables. Due to the exploratory nature of the study, models were adjusted for covariates which were selected *a priori* based on pain and knee OA literature. These included age, gender, BMI, OA symptom duration, baseline pain intensity and socioeconomic status (20, 114, 115). Baseline pain intensity was not included as a covariate in multivariable models at baseline where pain intensity measures were the dependent variables. Multicollinearity and heteroskedasticity were assessed for each analysis by examining multicollinearity statistics as well as scatterplots of residuals. The Durbin-Watson test was used to assess autocorrelation in the residuals with 1.5–2.5 being deemed acceptable (116). The level of error considered acceptable for statistical significance was set at *p* ≤ 0.05.

## 3. Results

### 3.1. Participant characteristics

**Figure 2:**
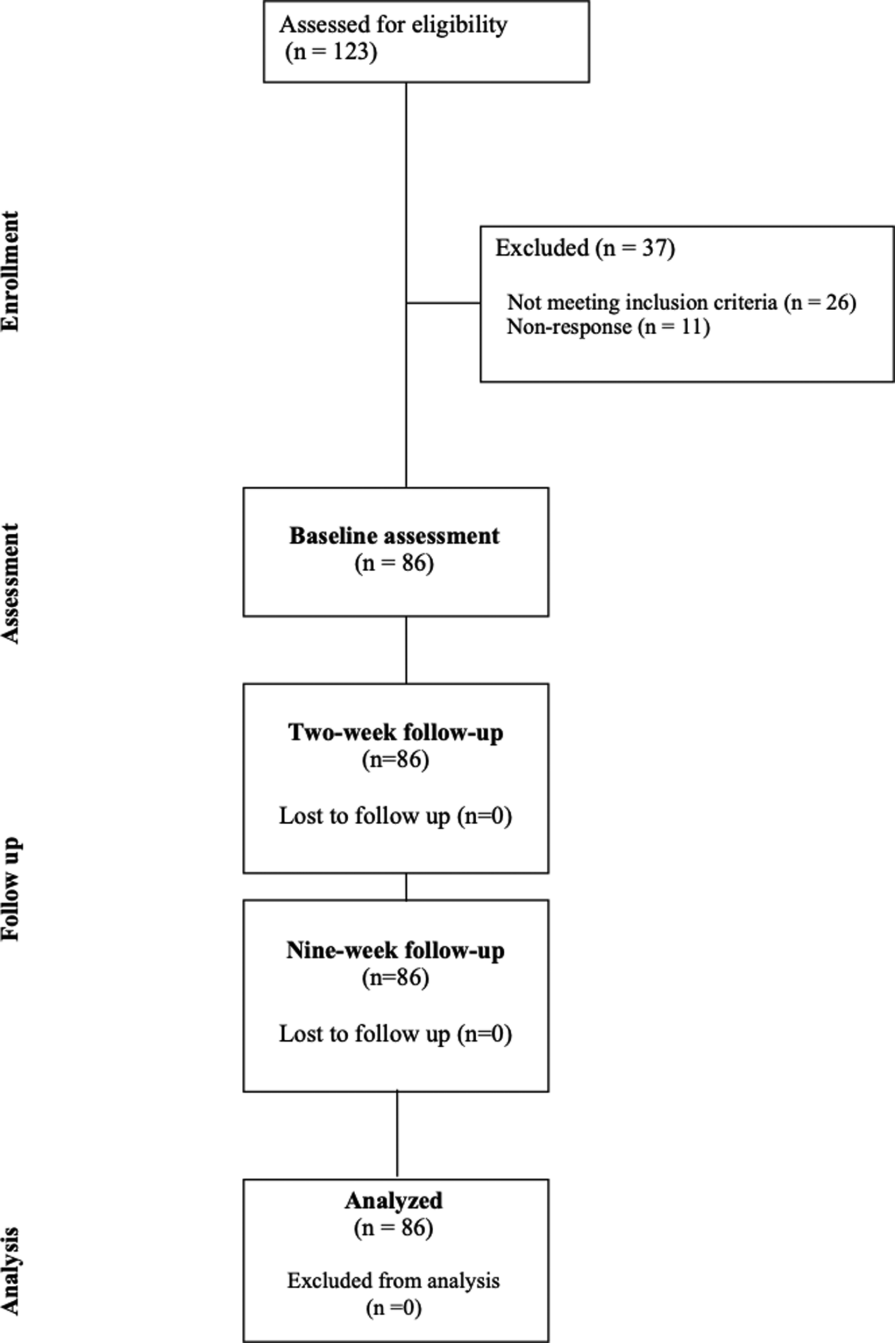
Participant flow diagram.

One hundred and twenty-three individuals registered to participate in the study. Twenty-six (21.1%) did not meet the inclusion criteria and were excluded. A further 11 participants (8.9%) did not respond to initial contact attempts. A total of 86 participants met the inclusion criteria and there was no loss to follow-up. This can be seen in the participant flow diagram in Figure 2.

The study sample is described in Table 2. Table 2 also provides means and standard deviations for baseline measures of included participants including participant characteristics, knee pain and functioning measures, psychosocial measures and health and lifestyle measures respectively.

**Table 2:** Baseline measures of included participants^a^

Characteristic	Value
Age (years)	67.3 ± 9.1
Sex [*n* (%)]	Female: 55 [64] Male: 31 [36]
Ethnicity [*n* (%)]	NZ European: 78 [90.7] Māori: 4 [4.7] Indian: 2 [2.3] English European: 1 [1.2] Egyptian: 1 [1.2]
BMI (kg/m^2^)	32 ± 6.8
Waist Hip Ratio [*n* (%)]	Normal: 1 [1.2] Overweight: 19 [22.1] Obese: 66 [76.7]
Handedness [*n* (%)]	Right: 80 [93] Left: 6 [7]
Highest level of education [*n* (%)]	No formal qualification: 12 [14] Year 10: 1 [1.2] Year 13: 12 [14] Trade/apprenticeship: 7 [8.1] Certificate/diploma: 19 [22.1] University degree: 19 [22.1] Postgraduate degree: 16 [18.6]
Work status [n (%)]	Fulltime employed: 21 [24.4] Part-time employed: 9 [10.5] Self-employed: 7 [8.1] Homemaker: 1 [1.2] Retired: 47 [54.7] Unable to work: 1 [1.2]
Knee pain and functioning measures	Value
Duration (years)	9.2 ± 9.1
Bilateral OA [*n* (%)]	Yes: 48 [55.8] No: 38 [44.2]
Worst knee [*n* (%)]	Right: 46 [53.5] Left: 40 [46.5]
Quad strength (kg/F)	Worst knee: 21.4 ± 7.4 Less affected knee: 23.9 ± 7.7
MQS	11.9 ± 10.8
Physical performance	6 MWT (m): 388.1 ± 90.8 30 sCST (n): 9.7 ± 2.9
BPI	Pain severity: 3.1 ± 1.6 Pain interference: 3.2 ± 2.3
Pain sites (n)	5 ± 3.8
ICOAP	Intermittent: 45.5 ± 20.7 Constant: 33.7 ± 24.8
KOOS	Pain: 55.6 ± 15.2 Symptoms: 55.4 ± 18.1 ADLs: 64.1 ± 18 Sports and recreation: 36.2 ± 23.3 QoL: 39.1 ± 19.7
Psychosocial measures	Value
DASS-21	Total: 10.86 ± 11.6 Depression: 3.7 ± 4.9 (Normal) Anxiety: 2.5 ± 3.3 (Normal) Stress: 4.7 ± 4.6 (Normal)
PSEQ-2	9.1 ± 2.6
PCS	12.1 ± 9.8
CSQ	36.2 ± 12.9
KOFBeQ:	56.5 ± 21.8
SPQ	16.4 ± 4.2
Health and lifestyle measures	Value
PSQI	8.3 ± 3.9
SF-12	34.1 ± 6.6
CCI:	1.2 ± 1.4
IPAQ [*n* (%)]	Low: 21 [24.4] Moderate: 22 [25.6] High: 43 [50]
Two-week follow-up measures	Value
BPI:	Pain severity: 3.1 ± 1.9 Pain interference: 2.8 ± 2.3
KOOS	Pain: 57.9 ± 15.3 Symptoms: 54.9 ± 18.1 ADLs: 66.1 ± 17.1 Sports and recreation: 40.5 ± 26.4 QoL: 41.2 ± 19.6
SF-12	34.6 ± 6
BFI	Fatigue severity: 4 ± 2.4 Fatigue interference: 3 ± 2.3
KAP	1.1 ± 1.5
Nine-week follow-up measures	Values
BPI:	Pain severity: 3.4 ± 1.8 Pain interference: 3 ± 2.3
KOOS	Pain: 57.5 ± 16.9 Symptoms: 54.9 ± 16.3 ADLs: 64.9 ± 18.9 Sports and recreation: 45.4 ± 27.6@ QoL: 39.9 ± 21.1

n, number; BMI, Body Mass Index; kg, kilograms; m, metres; OA, Osteoarthritis; F, Force; MQS, Medication Quantification Scale; 6 MWT, Six Minute Walk Test; m, metres; 30 sCST, 30-second Chair Stand Test; BPI, Brief Pain Inventory – short form, ICOAP, Measure of Intermittent and Constant Osteoarthritis Pain; KOOS, Knee Injury and Osteoarthritis Outcome Score; ADLs, Activities of Daily Living; QoL, Quality of Life; DASS-21, Depression, Anxiety, Stress Scale-21 item; PSEQ-2, Pain Self-efficacy Questionnaire – short form; PCS, Pain Catastrophising Scale; CSQ, Coping Strategies Questionnaire – short form; KOFBeQ, Knee Osteoarthritis Fears and Beliefs Questionnaire; SPQ, Social support and pain questionnaire; PSQI, Pittsburgh Sleep Quality Index; SF-12, Short-Form 12; CCI, Charleston Comorbidity Index; IPAQ, International Physical Activity Questionnaire – short form; BPF, Brief Fatigue Inventory; KAP, Keele Assessment of Participation.

^a^ Data are presented as mean ± standard deviation or number [%].

Table 3 provides means and standard deviations for sensitization measures.

**Table 3:** Summary of sensitization measures^a^

Sensitization measures	Value
PPI (NPRS)	Knee: 16.2 ± 19.1 Wrist: 10.1 ± 12.2
MTS (NPRS)	Knee: 18.4 ± 16.7 MTS: 34 [39.5] No MTS: 52 [60.5] Wrist: 15.6 ± 16.8
PPT (kPa)	Knee: 424.8 ± 258.4 Shin: 517.3 ± 244.4 Wrist: 447.4 ± 219
CPI (NPRS)	Knee: 25.6 ± 27.8 Wrist: 21.7 ± 27.7
CPM (% change)	1.6 ± 16.2 Inhibitor: 28 [32.6] Non-response: 30 [34.9] Facilitator: 28 [32.6]
MEP (NPRS)	6 MWT: 24.4 ± 18.8
SPA (NPRS)	6 MWT: 28.2 ± 24.2 SPA: 49 [57] No SPA: 37 [43] 30 sCST: 22.6 ± 25.6

MDT, Mechanical Detection Threshold; mN, millinewton; VDT, Vibration Detection Threshold, Hz, Hertz, MFC, medical femoral condyle; PPI, Punctate Pain Intensity; NPRS, Numeric Pain Rating Scale; MTS, Mechanical Temporal Summation; PPT, Pressure Pain Threshold; kPa, kilopascal; CPI, Cold Pain Intensity; CPM, Conditioned Pain Modulation; MEP, Movement-evoked pain; 6 MWT, Six Minute Walk Test; SPA, Sensitivity to Physical Activity; 30 sCST, 30-second Chair Stand Test.

^a^ Data are presented as mean ± standard deviation or number (%).

### 3.2. Longitudinal measures

Table 2 provides means and standard deviations for outcome measures collected at two-week and nine-week follow-up.

### 3.3. Univariable regression and correlation analyses

Results from the univariable regression and correlation analyses with *p*-values equal to or less than 0.2 are presented in Table 4.

**Table 4:** Unadjusted univariable regression and correlation outcomes between measures of sensitization and knee OA outcomes at baseline

Outcome variable	Predictor variable	*r*	*R* ^2^	*p*
BPI – pain intensity				
	MEP (6 MWT)	0.43	0.18	<0.001***
	Pain sites	0.33	0.11	0.002**
	SPA (6 MWT)	0.31	0.09	0.004**
	PPT (knee)	0.22	0.05	0.042*
	PPT (wrist)	0.22	0.05	0.044*
	PPT (shin)	0.18	0.03	0.094
BPI – pain interference				
	MEP (6 MWT)	0.28	0.08	0.009**
	SPA (6 MWT)	0.26	0.07	0.015*
	Pain sites	0.21	0.04	0.055
KOOS - pain				
	MEP (6 MWT)	0.51	0.27	<0.001***
	SPA (6 MWT)	0.45	0.20	<0.001***
	PPT (shin)	0.16	0.03	0.150
	PPT (knee)	0.15	0.02	0.178
KOOS - symptoms				
	MEP (6 MWT)	0.43	0.18	<0.001***
	SPA (6 MWT)	0.33	0.11	0.002**
	PPT (shin)	0.15	0.02	0.156
KOOS - ADLs				
	MEP (6 MWT)	0.48	0.23	<0.001***
	SPA (6 MWT)	0.36	0.13	<0.001***
	Pain sites	0.20	0.04	0.061
KOOS – Sports and recreation				
	MEP (6 MWT)	0.47	0.22	<0.001***
	SPA (30 sCST)	0.15	0.20	0.157
	SPA (6 MWT)	0.41	0.17	<0.001***
	PPT (knee)	0.20	0.04	0.066
	PPT (shin)	0.15	0.02	0.170
	Pain sites	0.18	0.03	0.102
KOOS - QoL				
	SPA (6 MWT)	0.30	0.09	0.006**
	MEP (6 MWT)	0.25	0.06	0.022*
	SPA (30 sCST)	0.16	0.03	0.147
	MTS (knee)	0.16	0.03	0.148
	CPM	0.15	0.02	0.183
ICOAP – constant				
	MEP (6 MWT)	0.28	0.08	0.009**
	SPA (6 MWT)	0.28	0.08	0.010**
	Pain sites	0.25	0.06	0.020*
ICOAP - intermittent				
	MTS (wrist)	0.29	0.09	0.006**
	MEP (6 MWT)	0.22	0.05	0.044*
	PPI (wrist)	0.20	0.04	0.068
	SPA (6 MWT)	0.17	0.03	0.129
	MTS (knee)	0.21	0.03	0.055
	PPI (knee)	0.17	0.03	0.110
	CPI (wrist)	0.18	0.03	0.096
SF-12				
	MEP (6 MWT)	0.32	0.10	0.003**
	SPA (6 MWT)	0.25	0.06	0.021*
	Pain sites	0.18	0.03	0.095
6 MWT				
	MEP (6 MWT)	0.45	0.20	<0.001***
	SPA (6 MWT)	0.44	0.20	<0.001***
	PPT (knee)	0.26	0.07	0.015*
	Pain sites	0.27	0.07	0.012*
	PPI (knee)	0.24	0.06	0.025*
	PPT (wrist)	0.22	0.05	0.041*
	PPT (shin)	0.21	0.05	0.048*
	MTS (wrist)	0.19	0.04	0.074
30 sCST				
	MEP (6 MWT)	0.34	0.12	0.001***
	PPI (knee)	0.31	0.09	0.004**
	SPA (6 MWT)	0.28	0.08	0.010**
	MTS (wrist)	0.28	0.08	0.010**
	Pain sites	0.28	0.08	0.010**
	MTS (knee)	0.22	0.05	0.055
	PPT (knee)	0.21	0.05	0.048*
	CPI (knee)	0.21	0.05	0.049*
	PPI (wrist)	0.21	0.04	0.041*
	CPI (wrist)	0.10	0.04	0.083
	PPT (wrist)	0.16	0.03	0.141
	SPA (30 sCST)	0.15	0.02	0.180

BPI, Brief Pain Inventory – short form; MEP, Movement-evoked pain; 6 MWT, Six Minute Walk Test; SPA, Sensitivity to Physical Activity; PPT, Pressure Pain Threshold; KOOS, Knee Injury and Osteoarthritis Outcome Score; ADLs, Activities of Daily Living; QoL, Quality of Life; MTS, Mechanical Temporal Summation; CPM, Conditioned Pain Modulation; ICOAP, Measure of Intermittent and Constant Osteoarthritis Pain; PPI, Punctate Pain Intensity; CPI, Cold Pain Intensity; SF-12, Short-Form 12; 30 sCST, 30-second Chair Stand Test.

*p ≤ 0.05; **p ≤ 0.01; ***p ≤ 0.001.

Bivariate correlations showed that MEP was significantly related with a number of outcomes. Similarly, Sensitivity to Physical Activity was also related with several outcomes. QST measures of sensitization demonstrated small to fair statistically significant correlations.

### 3.4. Multivariable regression analyses

Outcomes from the multivariable regression analyses between various measures of sensitization and outcomes presenting statistically significant at the 0.05 level cross-sectionally and longitudinally are presented in Tables 5, 6, respectively. Separate multivariable models were conducted for each predictor variable with adjustment for covariates including age, gender, BMI, knee OA duration, baseline pain intensity as well as socioeconomic status.

**Table 5:** Adjusted models of cross-sectional associations between measures of sensitization and clinical outcomes at baseline

Outcome variable	Predictor variable^b^	ß	*t*	*R* ^2^	*F* (*p*-value - model)
BPI – pain severity^a^					
	MEP (6 MWT)	0.04	3.80***	0.21	3.54 (0.004)
	Pain sites	0.13	2.75**	0.15	2.31 (0.042)
	SPA (6 MWT)	0.02	2.64**	0.14	2.20 (0.050)
KOOS - pain^a^					
	MEP (6 MWT)	−0.42	−5.03***	0.27	4.77 (<0.001)
	SPA (6 MWT)	−0.28	−0.44***	0.20	3.37 (0.005)
KOOS - symptoms					
	MEP (6 MWT)	−0.41	−4.01***	0.27	4.19 (<0.001)
	SPA (6 MWT)	−0.26	−3.28**	0.23	3.32 (0.004)
KOOS - ADLs					
	MEP (6 MWT)	−0.33	−3.69***	0.42	7.94 (<0.001)
	SPA (6 MWT)	−0.17	−2.34*	0.36	6.24 (<0.001)
KOOS - Sports					
	MEP (6 MWT)	−0.46	−3.60***	0.31	4.96 (<0.001)
	SPA (6 MWT)	−0.30	−3.00**	0.28	4.25 (<0.001)
SF-12					
	MEP (6 MWT)	−0.08	−2.05*	0.29	4.48 (<0.001)
6 MWT					
	MEP (6 MWT)	−1.46	−3.07**	0.37	6.54 (<0.001)
	SPA (6 MWT)	−1.12	−3.08**	0.37	6.55 (<0.001)
	PPT (knee)	0.10	2.30*	0.34	6.70 (<0.001)
	Pain sites	−5.17	−1.24*	0.33	5.58 (<0.001)
30 sCST					
	MTS (wrist)	−0.05	−2.54*	0.20	2.70 (0.014)
	MEP (6 MWT)	−0.05	−2.64**	0.20	2.80 (0.012)
	Pain sites	−0.19	−2.09*	0.18	2.37 (0.030)
	PPI (knee)	−0.03	−2.02*	0.17	2.32 (0.033)

BPI, Brief Pain Inventory – short form; MEP, Movement-evoked pain; 6 MWT, Six Minute Walk Test; SPA, Sensitivity to Physical Activity; KOOS, Knee Injury and Osteoarthritis Outcome Score; ADLs, Activities of Daily Living; SF-12, Short-Form 12; PPT, Pressure Pain Threshold; MTS, Mechanical Temporal Summation; PPI, Punctate Pain Intensity; 30 sCST, 30-second Chair Stand Test.

^a^ Baseline pain intensity not included as covariate in model.

^b^ Separate models for each predictor.

*p ≤ 0.05; **p ≤ 0.01; ***p ≤ 0.001.

**Table 6:** Adjusted models of associations between measures of sensitization and clinical outcomes at two- and nine-week follow-ups

Outcome variable	Predictor variable^a^	ß	*t*	*R* ^2^	*F* (model *p*-value)
BPI – pain severity (2-week)					
	MEP (6 MWT)	0.03	2.70**	0.45	9.00 (<0.001)
KOOS – pain (2-week)					
	MEP (6 MWT)	−0.32	−4.14***	0.43	8.55 (<0.001)
	SPA (6 MWT)	−0.17	−2.82**	0.37	6.64 (<0.001)
KOOS – symptoms (2-week)					
	MEP (6 MWT)	−0.39	−4.05***	0.34	5.60 (<0.001)
	SPA (6 MWT)	−0.23	−3.00**	0.28	4.29 (<0.001)
KOOS – ADLs (2-week)					
	MEP (6 MWT)	−0.28	−3.15**	0.39	6.77 (<0.001)
KOOS – sport (2-week)					
	MEP (6 MWT)	−0.51	−3.34**	0.23	3.31 (0.004)
	SPA (6 MWT)	−0.25	−2.05*	0.16	2.18 (0.045)
KOOS – QoL (2-week)					
	MEP (6 MWT)	−0.21	−2.00*	0.34	5.75 (<0.001)
	CPM	−0.37	−3.39**	0.40	7.30 (<0.001)
SF-12 (2-week)					
	MEP (6 MWT)	−0.09	−2.62*	0.30	4.87 (<0.001)
	PPT (wrist)	0.07	2.14*	0.29	4.44 (<0.001)
BFI – fatigue severity (2-week)					
	MEP (6 MWT)	0.04	3.17**	0.31	5.06 (<0.001)
BPI – pain severity (9-week)					
	MEP (6 MWT)	0.03	3.25**	0.39	7.25 (<0.001)
	SPA (6 MWT)	0.02	2.72**	0.37	6.60 (<0.001)
KOOS – pain (9-week)					
	MEP (6 MWT)	−0.32	−3.57***	0.37	6.50 (<0.001)
	SPA (6 MWT)	−0.19	−2.63**	0.33	5.38 (<0.001)
KOOS – symptoms (9-week)					
	MEP (6 MWT)	−0.29	−3.44**	0.29	4.45 (<0.001)
	SPA (6 MWT)	−0.19	−2.63**	0.26	3.93 (0.001)
KOOS – ADLs (9-week)					
	MEP (6 MWT)	−0.32	−2.99**	0.28	4.24 (<0.001)
	SPA (6 MWT)	−0.18	−2.11*	0.24	3.44 (0.003)
KOOS – sport (9-week)					
	MEP (6 MWT)	−0.44	−2.64**	0.17	2.23 (0.040)
	SPA (6 MWT)	−0.32	−2.53*	0.16	2.15 (0.048)
KOOS – QoL (9-week)					
	CPM	−0.29	−2.25*	0.26	3.93 (0.001)

Brief Pain Inventory – short form; MEP, Movement-evoked pain; 6 MWT; Six Minute Walk Test; KOOS, Knee Injury and Osteoarthritis Outcome Score; SPA, Sensitivity to Physical Activity; ADLs, Activities of Daily Living; QoL, Quality of Life; SF-12, Short-Form 12; BFI, Brief Fatigue Inventory; CPM, Conditioned Pain Modulation.

^a^ separate models for each predictor.

*p ≤ 0.05; **p ≤ 0.01; ***p ≤ 0.001.

At baseline, adjusted models including MEP explained a significant degree of variance across several outcomes including BPI-pain intensity (ß 0.04, *R*^2^ 21%), KOOS pain (ß −-0.42, *R*^2^ 27%), KOOS sport and recreation (ß −0.46, *R*^2^ 31%), 6 MWT performance (ß −1.46, *R*^2^ 37%) and health-related quality of life (ß −0.08, *R*^2^ 29%). SPA also explained a significant degree of variance in outcomes including KOOS pain (ß −0.28, *R*^2^ 20%), KOOS ADLs (ß −0.17, *R*^2^ 36%), KOOS sport and recreation (ß −0.30, *R*^2^ 28%) and 6 MWT performance (ß −1.12, *R*^2^ 37%). Adjusted models including pressure pain thresholds at the knee explained 34% of the variance in 6 MWT performance (ß 0.10) while MTS at the wrist explained 20% of 30 sCST performance (ß −0.05).

Adjusted models each including MEP and SPA continued to explain outcome variance at the two-week follow-up. MEP was shown responsible for variance in BPI – pain severity (ß 0.03, *R*^2^ 45%), KOOS pain (ß −0.32, *R*^2^ 43%), KOOS ADLs (ß −0.28, *R*^2^ 39%), health-related quality of life (ß −0.21, *R*^2^ 30%) and fatigue severity (ß 0.04, *R*^2^ 31%). SPA also explained outcome variance in KOOS pain (ß −0.17, *R*^2^ 37%), KOOS symptoms (ß −0.23, *R*^2^ 28%) and KOOS sports (ß −0.25, *R*^2^ 16%). At the two-week follow-up, CPM was shown to predict KOOS QoL outcomes explaining 40% of the variance (ß −0.37).

At the 9-week follow-up, adjusted models including MEP remained significant in explaining variance in BPI - pain severity (ß 0.03, *R*^2^ 39%), KOOS pain (ß −0.32, *R*^2^ 37%), KOOS ADLs (ß −0.32, *R*^2^ 28%) and KOOS sports (ß −0.44, *R*^2^ 17%). SPA continued to explain 37% of the variance in pain intensity at the 9-week follow-up (ß 0.02) as well as KOOS pain (ß −019, *R*^2^ 33%), KOOS symptoms (ß −0.19, *R*^2^ 26%), KOOS ADLs (ß −0.18, *R*^2^ 24%) and KOOS sports (ß −0.32, *R*^2^ 16%). Adjusted models including CPM continued to explain 26% of the variance in KOOS QoL outcomes (ß −0.29).

## 4. Discussion

Underlying pain mechanisms including nervous system sensitization have emerged as important considerations in musculoskeletal and pain research over recent decades. Activity-related pain measures, with links to nervous system sensitization, are also being more widely investigated (24, 29). This study explored whether these measures prospectively predict outcomes in those with knee OA. Findings included activity-related pain measures, such as MEP and SPA, demonstrated predictive associations with pain, function and health-related quality of life outcomes cross-sectionally as well as longitudinally, even after controlling for age, gender, BMI, symptom duration, socioeconomic status and baseline pain intensity. Therefore, people with knee OA who demonstrate greater activity-related pain may be at risk of higher pain, disability and reduced quality of life in a limited prospective period.

Both pain and disability are commonly reported by those suffering from knee OA (23). Therefore, measuring pain during functioning is highly relevant to the knee OA pain experience (24, 28). MEP is an emerging measure of activity-related pain which is represented by the average pain intensity reported during a standardized physical task. Therefore, MEP likely provides greater ecological validity to living and functioning with pain related to knee OA (24, 28, 117). Mechanisms of MEP are proposed to include peripheral mechanical factors including activation of silent nociceptors, as well as central nervous system changes resulting in lowered nociceptive thresholds (24, 28). This measure demonstrated predictive associations with distinct outcomes in the current study (i.e., health-related quality of life). Mechanisms of MEP may therefore be different from SPA and potentially include other related factors, such as psychosocial status which have been shown as important contributors towards health-related quality of life outcomes (118).

A recent study, which compared MEP with QST measures found that 12% of the variance of MEP was explained by TS, a marker of central sensitization (32). Therefore, at least some part of the underlying pain mechanisms of MEP involves central nervous system nociceptive changes reflective of central sensitization. Additionally, psychosocial factors, genetic and environmental factors are proposed to influence MEP (24, 28). Recent cross-sectional studies have confirmed that higher fear avoidance, pain catastrophizing and stress were related to greater MEP (23, 117). Interestingly, positive psychosocial factors, such as resilience have been found to demonstrate a protective buffering effect (23, 117). Therefore, addressing psychosocial factors could influence MEP and potentially improve outcomes.

MEP also has clinical importance with this activity-related measure potentially helping health professionals better understand the relationship between pain and functioning in those with knee OA (24). A recent prediction study assessed MEP using weight-bearing items from the Western Ontario and McMaster Universities Arthritis Index (WOMAC). This found that those with greater MEP prior to TKA had greater post-operative pain at one year (31). The current study also highlights that MEP importantly predicts future pain and disability outcomes in those living with knee OA in the community. Therefore, MEP may be an ecologically valid and clinically feasible measure of activity-related pain in those with knee OA.

Another activity-related measure of pain which captures the change in pain intensity during a physical task is SPA (30). Previous studies have highlighted the potential importance of SPA, with SPA cross-sectionally predicting pain and functional outcomes in those with knee OA (29, 30, 71, 119). Similar to MEP, underlying mechanisms of SPA are reported to include temporal summation as a result of the repetitive mechanical demands of physical tasks resulting in “wind-up” of nociception at the dorsal horn of the spinal cord (30, 70, 119–121). Studies have highlighted the impact that psychosocial factors, such as fear and pain catastrophising, have on SPA in those with knee OA (29, 30, 71, 119). Therefore, complex interactions between mechanical loading, pain mechanisms and psychosocial factors, and SPA are present in those with knee OA (29, 30, 71, 119).

Activity-related pain in the current study was assessed during OARSI-recommended physical performance measures including the 6 MWT and 30 sCST (69). The 6 MWT is a valid and reliable assessment of submaximal cardiovascular capacity (30, 122). An additional mechanism of activity-related pain could involve submaximal cardiovascular demands, which have been linked to the reactivity of the autonomic nervous system (123). The autonomic nervous system has been shown to have extensive interactions with nociceptive processing meaning that activation may result in greater nociception, reduced modulation and increased pain (124). This may explain why SPA and MEP during the 30 sCST, which arguably involves greater loading of the affected knee, had a limited predictive capacity in comparison to the 6 MWT in the current study. An impaired endogenous analgesia response to exercise (exercise-induced hypoalgesia), may also contribute to activity-related pain (125). As noted previously, psychosocial factors including pain-related fear, low mood and self-efficacy as well as pain catastrophizing have been independently linked to abnormal nociceptive processing and may influence pain experienced during physical activities (126–129). Therefore, mechanisms underlying activity-related pain are likely to be multifactorial and individual, including but not limited to mechanical, cardiovascular, autonomic, neural as well as psychological. Activity-related pain warrants consideration in clinical practice as it may act as a barrier to recommended exercise-based treatments (130), placing those with knee OA at risk of a negative cascade of sedentariness, disability and ongoing pain (29, 119). Further research is required to explore the mechanisms of activity-related pain and whether interventions such as pain self-management support including pain education, strategy implementation and exercise may be beneficial for this population (131–133).

QST is commonly used in clinical pain studies as a way of quantifying nervous system processing of noxious information (53). Previous studies have demonstrated the ability of QST to predict treatment outcomes in those suffering from musculoskeletal pain, highlighting that underlying pain mechanisms are an important consideration (20). Interestingly, in the current study, QST demonstrated variable relationships with pain outcomes. Cross-sectionally, PPTs at the wrist and knee demonstrated small correlations with pain intensity and 6 MWT performance. While CPI at the affected knee was not related with any outcomes. MTS, a lab-based psychophysical measure of central sensitization, was related with intermittent pain experiences, health-related quality of life and performance on the 30 sCST test. However, none of these relationships were maintained longitudinally.

When looking at the predictive capacity of QST, many of the measures did not meet the criteria for inclusion in multivariable models. However, in those that did, PPTs were shown to uniquely predict walking distance on the 6 MWT while greater MTS uniquely predicted worse performance on the 30 sCST, but not the 6 MWT. These findings potentially highlight some task-specific variability. Interestingly, CPM a measure of central nervous system pain modulation, demonstrated predictive associations with KOOS QoL outcomes longitudinally. These variable findings may be due to the range of different pain mechanisms presenting across the knee OA population, the susceptibility of QST being influenced by demographic and other factors, as well as the community sample which was made up by a majority experiencing a mild pain intensity (134). Further research exploring the predictive utility of QST measures in community samples of those with knee OA are needed to better support the use of these sensory measures in clinical practice.

Better understanding pain mechanisms and their ability to inform prognosis in those with knee OA could guide mechanism-based care and improve outcomes. By better understanding mechanisms involved in knee pain, targeted therapies could be provided that specifically focus on addressing the underlying cause of symptoms. A core recommended treatment for those with knee OA includes the prescription of physical activity (130). However, clinicians should be mindful that 57% of people with knee OA have SPA as demonstrated by this study. A more refined approach to activity prescription which considers activity-related pain and central pain mechanisms could aid in improving pain and functioning outcomes in the knee OA population while reducing the risk of symptom flares and subsequent fear avoidance and disability. Additionally, future research which considers mediating and moderating factors to better understand neurophysiological and biopsychosocial factors involved in knee OA pain would also provide useful information on targets for treatment (i.e., psychological interventions). Studies which use more ecologically valid biopsychosocial pain-related outcome variables with reduced recall bias, such as Ecological Momentary Assessment are warranted.

Strengths of the current study include both the cross-sectional and longitudinal design with follow-up data collected at two and nine weeks in a community sample of those living with knee OA. In addition, a range of measures assessing for sensitization were performed including static and dynamic QST as well as activity-related pain measures. Considering all of the above, this study provides a unique contribution to the pain mechanisms and knee OA literature, highlighting important prognostic markers in the community sample. Limitations include the majority of the sample reporting mild knee OA pain. Participants also lacked ethnic diversity with most being New Zealand European. Although one of the recruitment strategies was to recruit patients from an outpatient tertiary hospital setting, no participants were recruited *via* this route. The follow-up window of the current study was only nine weeks. Although this provides a brief longitudinal indicator as used by other knee OA studies (135), pain outcomes explored over at least 1–2 years would be useful to determine the medium- and long-term trajectories and outcomes. Participants within the current study may present as less sensitized compared to other samples (average remote PPTs were 447.4 ± 219 kPa compared to 146–369 kPa in the literature) (11). Scores on psychological measures were also low in the current sample meaning the included sample may not fully represent the wider knee OA population. Psychometric properties of MEP and SPA measures also need to be established including exploration of MEP and SPA during unstandardized, free-living functional tasks to maximise ecological validity. Variable selection methods for including variables in the multivariable analysis may have wrongly rejected potentially important candidate variables, however, a larger *p*-value threshold was used to reduce the risk of this. Furthermore, multivariable model checks were completed for candidate variables from the univariable analysis with no additional predictor variables meeting statistical significance (Appendix A). Overall, larger, multi-centred studies of people with moderate and severe knee OA with longer follow-up durations are required. External validation of the models exploring potentially important moderators and mediators of these relationships is warranted.

## 5. Conclusion

Pain and reduced functioning remain a major problem, contributing to disability and reduced quality of life in those with knee OA. Alterations in underlying nervous system pain mechanisms have been highlighted in this population which may inform prognosis. This was confirmed by the current study which highlights that activity-related pain measures including MEP and SPA, demonstrate predictive associations with pain intensity, function and quality of life outcomes prospectively in those with knee OA. Pain mechanisms assessed *via* QST demonstrated variable relationships with outcomes which were often not maintained longitudinally.

Therefore, consideration of clinically feasible, ecologically valid measures of activity-related pain and sensitization is recommended when assessing and treating those with knee OA. This could provide important clinical information and assist in identifying those at greatest risk of pain and disability. By identifying those at higher risk, treatments can be provided to those in greatest need and potentially target mechanisms of activity-related pain to improve knee OA outcomes. However, before these measures are routinely implemented into clinical practice, further studies are required that externally validate findings as well as explore measures of activity-related pain over longer durations, in different samples and use a range of standardized physical tests.

## Data Availability

The raw data supporting the conclusions of this article will be made available by the authors, without undue reservation.

## APPENDIX A Multivariable candidates not meeting univariable selection criteria.

**Table:**

Outcome Variable	Predictor Variable	*R*	*R* ^2^	Multivariable *p*-value	Univariable *p*-value
BPI – pain intensity					
	MTS (knee)	0.34	0.12	0.20	0.26
	PPI (knee)	0.26	0.07	0.92	0.48
	MTS (wrist)	0.28	0.08	0.34	0.69
	CPI (knee)	0.26	0.07	0.93	0.40
	CPM	0.28	0.08	0.33	0.33
BPI – pain interference					
	MTS (knee)	0.25	0.06	0.11	0.21
	PPI (knee)	0.23	0.05	0.20	0.41
	MTS (wrist)	0.20	0.04	0.45	0.50
	PPI (wrist)	0.21	0.04	0.82	0.38
	PPT (knee)	0.18	0.03	0.72	0.51
	PPT (wrist)	0.18	0.03	0.80	0.98
	CPM	0.18	0.03	0.80	0.74
ICOAP -constant					
	MTS (knee)	0.26	0.07	0.06	0.22
	PPI (knee)	0.18	0.03	0.37	0.68
	MTS (wrist)	0.21	0.04	0.18	0.31
	PPI (wrist)	0.25	0.06	0.07	0.25
	PPT (knee)	0.17	0.03	0.47	0.23
	PPT (wrist)	0.16	0.02	0.62	0.40
ICOAP – intermittent					
	PPT (knee)	0.18	0.03	0.77	0.65
	PPT (wrist)	0.18	0.03	0.73	0.73
	CPM	0.19	0.04	0.46	0.41
KOOS - symptoms					
	PPT (wrist)	0.23	0.05	0.26	0.33
KOOS - pain					
	MTS (wrist)	0.22	0.05	0.28	0.37
	PPT (wrist)	0.20	0.04	0.42	0.38
	CPM	0.21	0.04	0.33	0.39
KOOS - ADLs					
	MTS (knee)	0.33	0.11	0.23	0.62
	PPI (knee)	0.30	0.09	0.99	0.51
	MTS (wrist)	0.31	0.10	0.66	0.96
	PPT (knee)	0.33	0.11	0.29	0.40
KOOS – sports					
	PPI (knee)	0.33	0.11	0.79	0.24
	CPM	0.33	0.11	0.57	0.46
KOOS - QoL					
	MTS (wrist)	0.32	0.10	0.16	0.28
	PPT (knee)	0.28	0.08	0.73	0.99
	CPI (knee)	0.31	0.10	0.21	0.32

BPI, Brief Pain Inventory – short form; MTS, Mechanical Temporal Summation; PPI, Punctate Pain Intensity; CPI, Cold Pain Intensity; CPM, Conditioned Pain Modulation; PPT, Pressure Pain Threshold; ICOAP, Measure of Intermittent and Constant Osteoarthritis Pain; KOOS, Knee Injury and Osteoarthritis Outcome Score; ADLs, Activities of Daily Living; QoL, Quality of Life.
